# *atoh8* expression pattern in early zebrafish embryonic development

**DOI:** 10.1007/s00418-021-02001-z

**Published:** 2021-06-13

**Authors:** Ninfa Fragale, Satya Srirama Karthik Divvela, Beate Brand-Saberi

**Affiliations:** Department of Anatomy and Molecular Embryology, Institute of Anatomy, Ruhr-University, Bochum, Germany

**Keywords:** *atoh8*, Zebrafish, Somite, Sclerotome, Myotome, Hindbrain

## Abstract

Atonal homologue 8 (*atoh8*) is a basic helix-loop-helix transcription factor expressed in a variety of embryonic tissues. While several studies have implicated *atoh8* in various developmental pathways in other species, its role in zebrafish development remains uncertain. So far, no studies have dealt with an in-depth in situ analysis of the tissue distribution of *atoh8* in embryonic zebrafish. We set out to pinpoint the exact location of *atoh8* expression in a detailed spatio-temporal analysis in zebrafish during the first 24 h of development (hpf). To our surprise, we observed transcription from pre-segmentation stages in the paraxial mesoderm and during the segmentation stages in the somitic sclerotome and not—as previously reported—in the myotome. With progressing maturation of the somites, the restriction of *atoh8* to the sclerotomal compartment became evident. Double in situ hybridisation with *atoh8* and *myoD* revealed that both genes are expressed in the somites at coinciding developmental stages; however, their domains do not spatially overlap. A second domain of *atoh8* expression emerged in the embryonic brain in the developing cerebellum and hindbrain. Here, we observed a specific expression pattern which was again in contrast to the previously published suggestion of *atoh8* transcription in neural crest cells. Our findings point towards a possible role of *atoh8* in sclerotome, cerebellum and hindbrain development. More importantly, the results of this expression analysis provide new insights into early sclerotome development in zebrafish—a field of research in developmental biology which has not received much attention so far.

## Introduction

Atonal homologue 8 (*atoh8*) is a basic helix-loop-helix (bHLH) transcription factor implicated in a multitude of developmental and physiological processes in health and disease. In zebrafish, its impact on organogenesis has been studied with respect to the formation of muscle tissue and the retina (Yao et al. 2010), swim bladder and heart (Place and Smith 2017; Rawnsley et al. 2013), and haematopoiesis and angiogenesis (Place and Smith 2017). Most of these studies have employed morpholinos—stabilised antisense RNA oligonucleotides—to transiently silence gene expression. Depending on the dose injected, these studies have produced different outcomes in the generated morphants. The only knockout study in zebrafish so far did not confirm a phenotypic effect due to loss of *atoh8*, leading to the conclusion it might be dispensable for zebrafish embryonic development (Place and Smith 2017).

Although several studies have suggested a role for *atoh8* in zebrafish embryonic development (Rawnsley et al. 2013; Yao et al. 2010; Fang et al. 2014), there has not been any detailed in situ hybridisation (ISH) analysis in zebrafish. Thus, the exact tissue location of zebrafish *atoh8* expression is still unknown. The first general description located *atoh8* transcription to somites, neural crest and the developing eye (Yao et al. 2010). Based on the observed expression profile, they investigated a role for *atoh8* in the developing retina and somites and proposed that *atoh8* regulated the formation of the retinal layers in the eye and of muscle tissue in the somites (Yao et al. 2010). Somites are transient metameric segments of the embryonic vertebrate body plan, which emerge from the paraxial mesoderm in a periodicity regulated by a molecular clock of oscillating gene expression (Bénazéraf and Pourquié 2013; Maroto et al. 2012; Stickney et al. 2000; Tajbakhsh and Spörle 1998). Somitogenesis and development in zebrafish follows the same pattern as in amniotes (Keenan and Currie 2019; Stickney et al. 2000; Brand-Saberi et al. 1996): the pre-segmental paraxial mesoderm is subdivided into repeated units, which then compartmentalise into dermomyotome, giving rise to skin and skeletal muscle, and sclerotome, forming the skeletal and associated connective tissue components of the axial skeleton. In zebrafish, somitic epithelial cells located next to the notochord, so-called adaxial cells (Thisse et al. 1993), are the first to express muscle-specific markers, specifically the bHLH transcription factor *myoD*, before the onset of overt segmentation and are the precursors of the slow fibre type muscles (Devoto et al. 1996; Weinberg et al. 1996). Most of the adaxial cells start migrating away from their medial position eventually giving rise to a superficial single layer of mononucleated slow muscle fibres. Fast and intermediate muscle tissue originates from the inner mesenchymal core of cells in the somite.

While somitogenesis and muscle development has been studied extensively in both amniotes (Monsoro-Burq 2003; Stockdale et al. 2000; Tani et al. 2020; Saga 2012; Gridley 2006; Brand-Saberi et al. 1996) and zebrafish (Devoto et al. 1996; Weinberg et al. 1996; Stellabotte et al. 2007; Nguyen-Chi et al. 2012), the formation of sclerotome and the genetic regulatory networks involved have remained understudied in zebrafish. A sclerotomal compartment in the ventral somite has been described in zebrafish, albeit in the context of the influence of sclerotome development on segmentation of the peripheral nervous system (Morin-Kensicki and Eisen 1997) and the vertebral column (Morin-Kensicki et al. 2002). In zebrafish, a contribution of sclerotome cells to the neural and haemal arches has been established; however, whether the sclerotome contributes to the formation of centra is still being disputed (Bensimon-Brito et al. 2012; Fleming et al. 2001, 2004, 2015; Inohaya et al. 2007; Renn et al. 2013; Willems et al. 2012). At the molecular level, several key markers have been identified for specification of sclerotome and its derivatives in amniotes. Among these are *twist*, *pax1*, *pax9*, *nkx3.1* and *nkx3.2* (Akazawa et al. 2000; Balling et al. 1996; Barnes and Firulli 2009; Gitelman 1997; Herbrand et al. 2002; Peters et al. 1999; Provot et al. 2006; Rainbow et al. 2014; Tribioli and Lufkin 1999). Expression of these markers has been shown to be conserved in the zebrafish sclerotome (Germanguz et al. 2007; Nornes et al. 1996; Liu et al. 2013; Ma et al. 2018), but only very rarely has their involvement in specification and development of this somitic compartment been investigated using this model organism (Yang et al. 2011; Ma et al. 2018; Chen et al. 2014).

A second domain of *atoh8* expression appears to be the neural crest (Yao et al. 2010). The neural crest is a transient, multipotent and migratory embryonic cell lineage located in the neural plate border along the anterior–posterior axis. Neural crest cells undergo epithelial-mesenchymal transition (EMT) and migrate to final destinations in the embryo (Rocha et al. 2020). The cephalic neural crest contributes to various elements of the head, such as bone and connective tissue of the jaw and skull, cranial sensory ganglia, peripheral neuronal and glial cells and melanocytes. The trunk neural crest constitutes a domain of cells arising along the extent of the spinal cord and generating neurons and glia of the dorsal root ganglia and autonomous nervous system as well as melanocytes and other cell types. In zebrafish, neural crest progenitors emerge from about 10.5 h post fertilisation (hpf) at the lateral margin of the neural ectoderm (Odenthal and Nüsslein-Volhard 1998; Thisse et al. 1995) and—once specified as the neural crest—start their journey in an anterior to posterior sequence. Cranial neural crest cells begin their migration at about 13 hpf in streams emanating from the midbrain and hindbrain regions and travelling anteriorly to contribute to the neurocranium, and ventrally and posteriorly to produce the viscerocranium of the pharyngeal arches (Schilling and Kimmel 1994). From 15 hpf, zebrafish trunk neural crest migrate—as in other vertebrates—in series of reiterated streams on two distinct pathways (Raible et al. 1992): the dorsal and the lateral pathways. Neural crest cells located at the lateral border of the developing neural tube enter the dorsal pathway and migrate between the somite and the neural tube. At a later stage, neural crest cells from the dorsal-most aspect of the neural tube migrate on the lateral pathway between the somite and the overlying ectoderm.

*atoh8* has so far not been associated with zebrafish cerebellum and hindbrain development, in contrast to its mouse homologue *math6*, which has been shown to be expressed in the developing central nervous system (Inoue et al. 2001). In zebrafish, as in mouse and chick, the hindbrain becomes transiently morphologically distinctive when eight metameric bulges—the rhombomeres (r)—form the neuroepithelium of the posterior brain (in zebrafish: from 18 to 20 hpf; Kimmel et al. 1995). The segmentation at the morphological level is paralleled by the molecular compartmentalisation of cells into units with differential and segment-specific gene expression, thus keeping apart cells of different lineages and biological functions (Kiecker and Lumsden 2005; Terriente et al. 2012). The morphological segregation of cell lineages during embryonic life, although lost during subsequent growth and cell migration, is of outmost importance for the neuroanatomical and functional organisation of the adult brain. One structure of particular interest in the developing hindbrain is the rhombic lip, which emerges transiently on the dorsal part of the rhombencephalon and provides a germinal zone for the different compartments of the mature posterior brain. The anterior-most region of the rhombic lip is referred to as the upper rhombic lip (URL) and coincides with the dorsal pole of r1. The URL generates the granule cells of the external and internal granular layers of the cerebellum (Volkmann et al. 2010, 2008). The lower rhombic lip (LRL) extends at the surface of r2 to r8 and provides the neuronal progenitor populations for the deep nuclei of the brainstem and the pre-cerebellar nuclei (Wullimann et al. 2011). Multiple proneural genes have been shown to be expressed in the zebrafish rhombic lip, among them members of the atonal subfamily of bHLH transcription factors, namely *atoh1a*, *atoh1b*, *atoh1c* and *atoh2*. These genes are expressed in overlapping but distinct domains in the rhombic lip of the zebrafish rhombencephalon and characterise progenitor cell populations contributing to the cerebellar granule cells and to specific deep nuclei of the brainstem (Adolf et al. 2004; Belzunce et al. 2020; Kani et al. 2010; Kidwell et al. 2018; Millimaki et al. 2007).

Based on previous reports of *atoh8* expression in zebrafish embryonic development (Yao et al. 2010), we conducted double in situ hybridisation of *atoh8*, *crestin*, a specific pan-neural crest marker (Luo et al. 2001) and *myoD,* the first of the muscle regulatory factors to be expressed in zebrafish (Weinberg et al. 1996), which revealed mutually exclusive expression domains for the three genes. As part of our study, we also examined the literature for expression patterns of sclerotome and neural crest key markers and found temporal and spatial overlap with *atoh8* in their profiles. So far, this is the first analysis of *atoh8* expression conducted in such detail, and it reveals a spatio-temporal profile far more complex than previously thought. Our results do not support the previous assumption that the location of *atoh8* transcription is the myotome (Yao et al. 2010), but clearly show that *atoh8* is expressed in the sclerotome compartment of the somite. This not only sheds new light onto how *atoh8* might be implicated in early developmental processes, but, more importantly, also advances investigations into the—in our view, neglected—area of sclerotome development in zebrafish.

## Material and methods

### Zebrafish care

Zebrafish (*Danio rerio*) were maintained at 28.5 °C on a 14 h light/10 h dark cycle. Embryos were collected and raised at 28.5 °C in E3 medium supplemented with 0.01% methylene blue to the desired stages.

### Staging

The developmental stages were determined according to Kimmel et al. (1995). All embryos were manually dechorionated and fixed for expression studies.

### Whole-mount in situ hybridisation

Whole-mount in situ hybridisation was performed following standard protocols (Thisse and Thisse 2014), however, using a hybridisation temperature of 60 °C. Briefly, embryos were manually dechorionated and fixed in 4% PFA/PBS overnight at 4 °C. Following dehydration in 50% and 100% methanol in PBS, the embryos were stored at –20 °C until further use. For ISH, embryos were rehydrated and post-fixed in 4% PFA/PBST for 20 min at room temperature (RT). Proteinase K digest (10 µg/ml in PBST) was performed for the duration appropriate to the stages of the embryos (Thisse and Thisse 2014) and stopped with 0.2 M glycine in PBS. Embryos were again post-fixed in 4% PFA/PBST for 20 min at RT, and then washed three times for 5 min in PBST at RT. Pre-hybridisation and hybridisation (in hybridisation solution without probes) was performed at 60 °C in the hybridisation oven (HO) for 2 h and overnight, respectively. Hybridisation with *atoh8* and *crestin* (DIG-labelled, in single staining) and *myoD* and *crestin* (FITC-labelled, in double staining) probes (1 μg/ml for each probe) was performed overnight at 60 °C (HO). After successive washes in 1:1 formamide in 2× SSC, 0.1% Tween-20 and in 0.2× SSC, 0.1% Tween-20 for 20 min at 60 °C, followed a blocking step for at least 4 h at RT. Thereafter the embryos were incubated in blocking buffer for at least 4 h at RT. For single staining, embryos were incubated in anti-digoxigenin-AB-Fab-fragments (Sigma-Aldrich, # 11093274910) in blocking solution (1:5000) overnight at 4 °C. In the double in situ hybridisation procedure, staining of the *atoh8* probe was performed first. We incubated the embryos in the dark for more than 6 h at RT in staining solution (20 µl/ml NBT/BCIP [Sigma-Aldrich, # 11681451001] in alkaline phosphatase [AP] buffer, pH 9.5) and in some instances, after two washes of 5 min in KTBT, we kept the embryos in fresh KTBT overnight gently shaking at 4 °C. The next day, after two washes in AP buffer, the staining reaction was continued for further 1–3 h at 30 °C (HO). Embryos were then washed three times for 10 min in KTBT at RT, and the blocking step was repeated, before applying the anti-FITC antibody overnight at 4 °C. Four washes for 25 min in KTBT and three washes for 5 min in AP buffer (pH 8.0) preceded the second staining reaction which was performed with SigmaFast™ Fast Red tablets (Sigma-Aldrich, # F4523) diluted 1:1 in AP buffer (pH 8.0). When staining reached the desired intensity, the reaction was stopped by two washes in KTBT for 10 min at RT. The embryos were subsequently washed in PBS (twice at 10 min, RT) and fixed in 4% PFA for storage. Embryos selected for imaging were incubated overnight in 100% glycerol at 4 °C. *crestin* was a gift from Marnie Halpern (Addgene plasmid # 89394; http://n2t.net/addgene:89394; RRID:Addgene_89394), (Rubinstein et al. 2000).

### Vibratome sections

In situ hybridised embryos were embedded in 3% agarose dissolved in PBS and cut at a thickness of 50 µm (*atoh8*) and 30 µm (*atoh8/myoD*, *atoh8/crestin*) on a Leica VT 1000 S vibratome. The sections were collected onto slides and mounted in Aquatex (Sigma-Aldrich, # V108562).

### Microscopy and imaging

After in situ hybridisation, whole embryos were observed under the Leica M165 FC microscope at ×12, ×10, and ×8 magnification and photographed using a Leica DFC420 C digital camera. Images from the vibratome sections were obtained at ×10, ×20 and, from the flat mount shown in Fig. 2h, at ×40 magnification using an Olympus BX61VS microscope (N.A. 0.40, 0.75 and 0.95, respectively;). The photos were further processed using Adobe Photoshop version 21.1.3.

### RNA Isolation, reverse transcription, and quantitative real-time PCR

For each of the analysed 2.5- to 11-hpf stages of development, 50 embryos were pooled and collected in TRI Reagent to detect *atoh8* expression. Next, RNA was isolated from the pooled embryos using the manufacturer’s instructions for TRI Reagent (Sigma-Aldrich, Munich, Germany). Following RNA isolation, the RNA concentration was determined, and 1 µg of RNA was reverse-transcribed to generate cDNA. Reverse transcription reaction was performed with GoScript reverse transcriptase (Promega), following the manufacturer’s instructions. The obtained cDNA was used as a template to perform real-time quantitative PCR (RT-qPCR). The RT-qPCR reaction was performed using the GoTaq qPCR master mix (Promega, Mannheim, Germany), following the manufacturer's instructions. The gene expression shown is the mean of three technical replicates which are normalised to *actin beta 2* (*actb2*). mRNA sequences used were: *atoh8* mRNA sequence PubMed accession no. NM_001079991.2 and *actb2* mRNA sequence PubMed accession no. NM_181601.5. Primers used were as follows:

*atoh8* 5ʹ-GCCATTCAGCAGACTCGGA-3ʹ; 5’-CTGCCCATAAGAGTAGCAGGG-3ʹ;

*actb2* 5ʹ -AAGGCCAACAGGGAAAAGAT-3ʹ; 5ʹ-AGGGCGTAACCCTCGTAGAT-3ʹ.

### Ethics approval

According to German regulations, the use of zebrafish in animal experiments needs approval only if the zebrafish is capable of independent feeding, which is the case from 120 hpf onwards. We limited our experiments and observations to the first 24 hpf of embryonic zebrafish; therefore no government approval or permission was required for this study.

## Results

### *atoh8* expression pattern in two phases

**Fig. 1:**
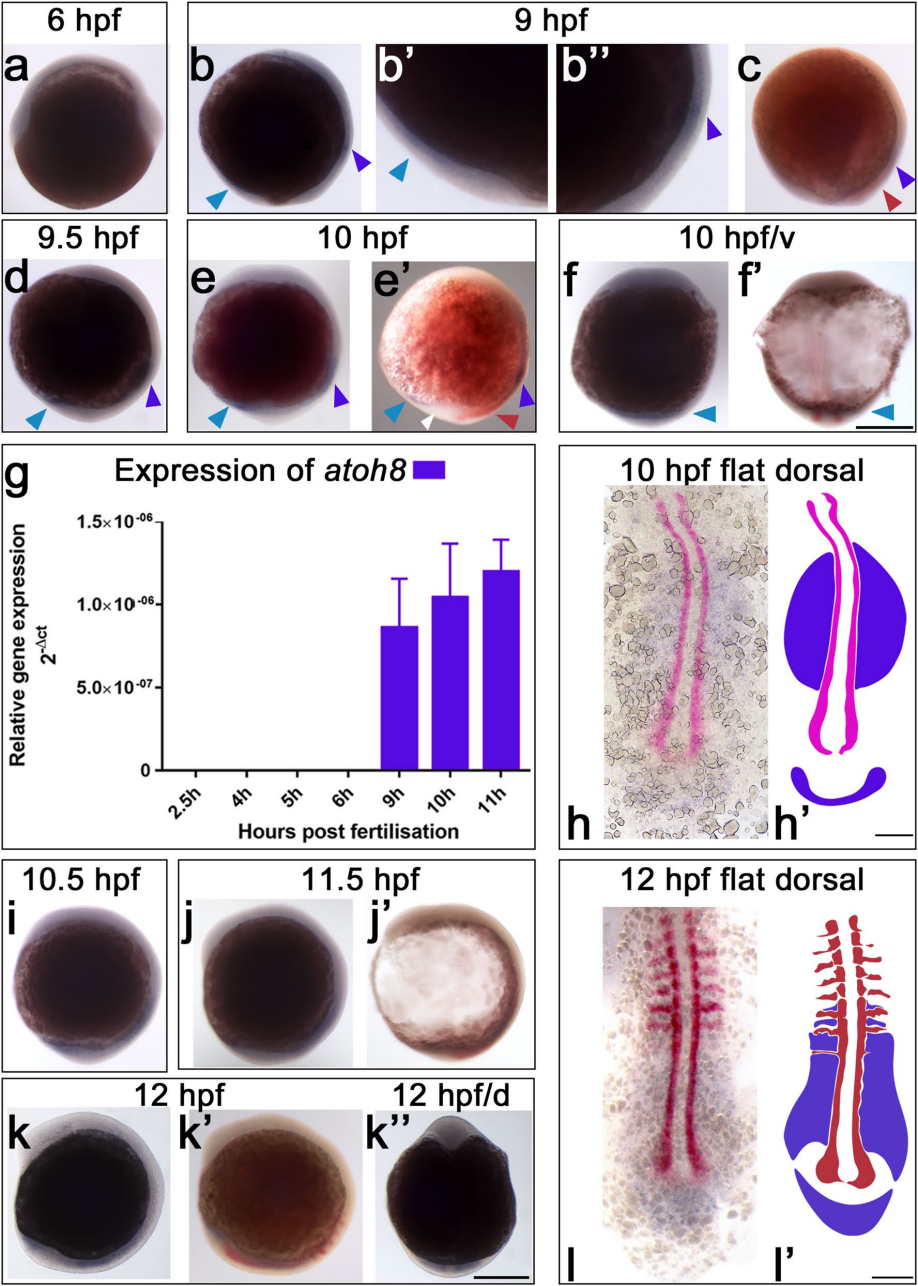
*atoh8* (blue) and *myoD* expression (red) in the gastrula and early segmentation stages of development (6–12 hpf). **a** Shield stage (6 hpf). **b** 90% epiboly (9 hpf). **b’**, **b’’** magnifications of image (**b**) showing dorsal and ventral *atoh8* expression, respectively. **c** 9-hpf embryo double-stained for *atoh8* and *myoD*. **d** *atoh8* expression at 9.5 hpf. **e**, **e’** 10-hpf embryos stained for *atoh8* alone and *atoh8* and *myoD*, respectively; white arrowhead in e' indicates tail bud tissue free of *atoh8* expression. **f**, **f’** 10-hpf embryos stained for *atoh8* alone and *atoh8* and *myoD*, respectively. **g** results of RT-qPCR for *atoh8* at developmental stages 2.5 to 11 hpf showing its expression relative to *actb2*. **h**, **h’** Flat mount and schematic drawing of 10-hpf embryo showing *atoh8* and *myoD* expression in blue and red, respectively. **i** *atoh8* staining at 9.5 hpf. **j**, **j’** 11.5-hpf embryos stained for *atoh8* alone and *atoh8* and *myoD*, respectively. **k**, **k’’** 12-hpf embryos stained for *atoh8*. **k’** 12-hpf embryo double-stained for *atoh8* and *myoD*. **l**, **l’** Flat mount and schematic drawing of 12-hpf embryo showing *atoh8* and *myoD* expression in blue and red, respectively. Whole embryos: lateral view, anterior to the top, dorsal to the right. **d’** ventral view (v); **f**, **h**, **k**, **l** dorsal view. Blue and red arrowheads: sites of *atoh8* and *myoD* expression, respectively. Scale bars 200 µm

**Fig. 2:**
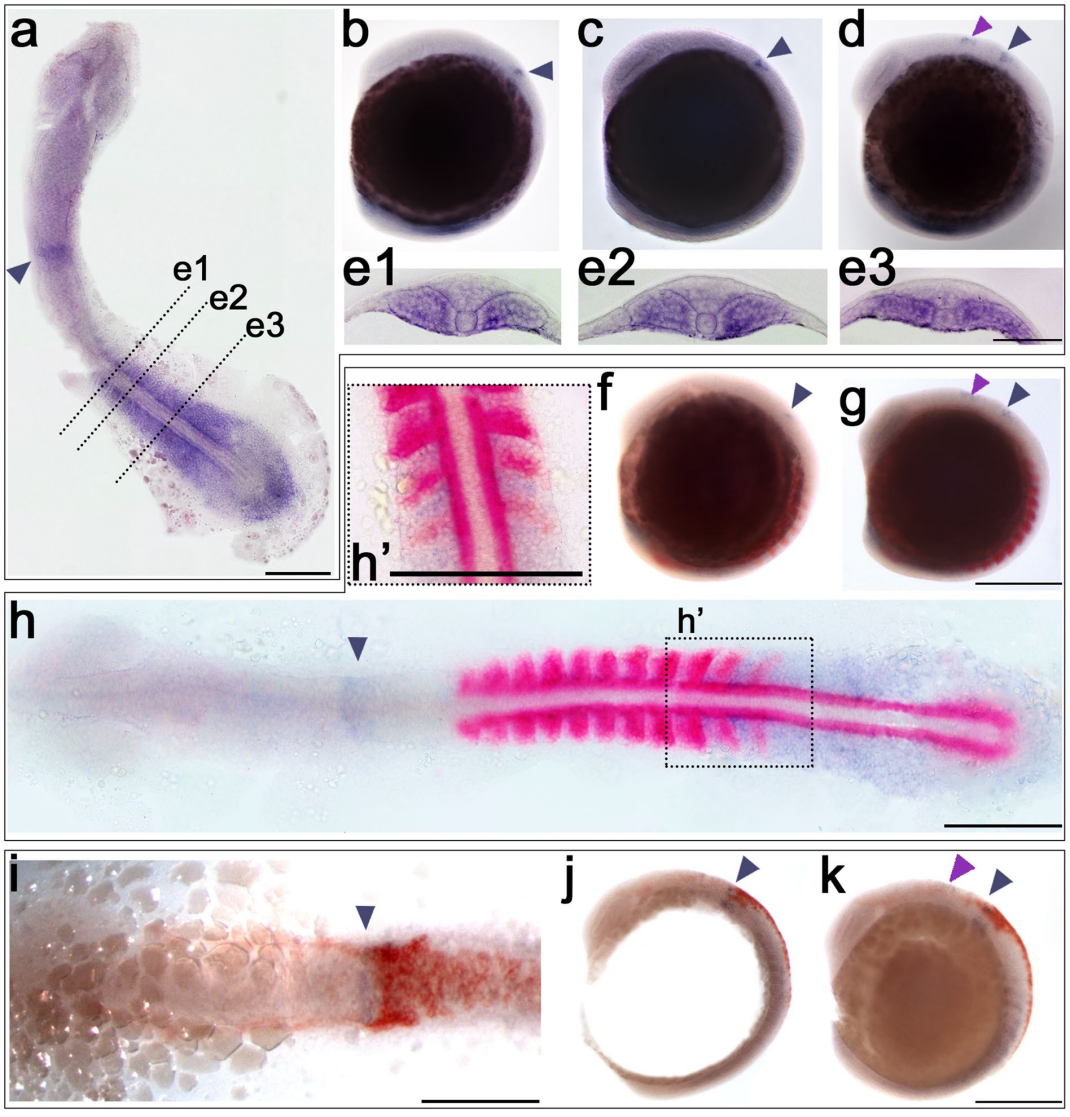
*atoh8*, *myoD* and *crestin* expression in early segmentation stages of development (13–14 hpf**)**. **a**–**e** *atoh8* ISH; **f**–**h** *atoh8/myoD* double ISH. **i**–**k** *atoh8/crestin* double ISH. **a** Flat mount of 13.5-hpf embryo. **b** 13-hpf embryo showing first appearance of *atoh8* in the developing hindbrain (dark arrowhead). **c** 13.5-hpf embryo. **d** Embryo at 14 hpf; pink arrowhead indicates the appearance of the second domain at the midbrain/hindbrain boundary. **e1**–**e3** Sections through the 13-hpf embryo at the levels indicated in **a**. **f**, **g** Embryos at 13 hpf and 14 hpf, respectively, double-stained for *atoh8* and *myoD*. **h** Flat mount of embryo at 14 hpf double-stained for *atoh8* and *myoD*. **i**, **j** Flat mount and embryo showing double labelling for *crestin* and *atoh8* at 13 hpf. **k** Embryo at 14 hpf double-stained for *crestin* and *atoh8*. **a**, **h’** Dorsal view, anterior to the top. **b**–**d**, **f**, **g**, **j**, **k** lateral view, anterior to the top, dorsal to the right. **h**, **i** dorsal view, anterior to the left. Scale bars: whole embryos 200 µm, flat mounts **a**, **h**, **i** 200 µm; **h’** 50 µm; transverse sections: 100 µm

**Fig. 3:**
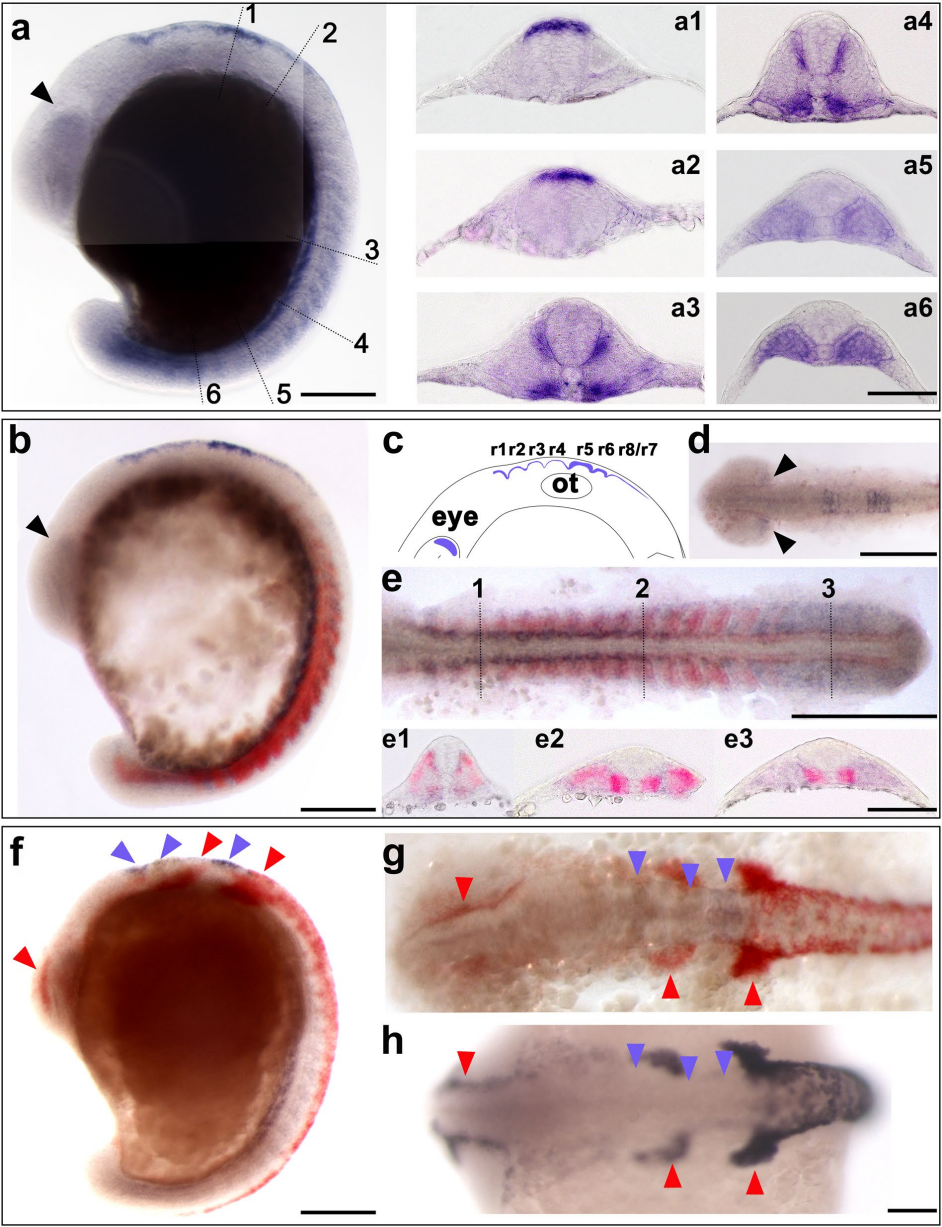
*atoh8*, *myoD* and *crestin* expression profile in the 16–16.5-hpf embryo. **a**, **b**, **f** Whole embryos at 16.5 hpf, showing *atoh8* and *atoh8/myoD* and *atoh8/crestin* signal distribution, respectively; lateral view, dorsal to the right. Arrowheads point at expression in areas of the developing eye, hindbrain and neural crest; anterior to the top. **a1**–**a6** Transverse sections of embryo in (**a**) at the respective levels; dorsal to the top. **c** Schematic diagram of *atoh8* distribution in brain tissue. **d** Flat mount of a 16-hpf embryo showing *atoh8* expression; dorsal view, anterior to the left. Flat mount (anterior to the left) of 16-hpf embryo double-stained for *atoh8* and *myoD*. **e**, **e1**–**e3** transverse sections (dorsal to the top) through levels indicated in **e**. **f**, **g** *atoh8/crestin* signal distribution; arrowheads indicating presence/absence of *atoh8* transcripts (violet) and *crestin* transcripts (red). **h** Whole embryo showing *crestin* expression in the head region; dorsal view, anterior to the left. Scale bars: whole embryos, flat mounts 200 µm; transverse sections 100 µm

**Fig. 4:**
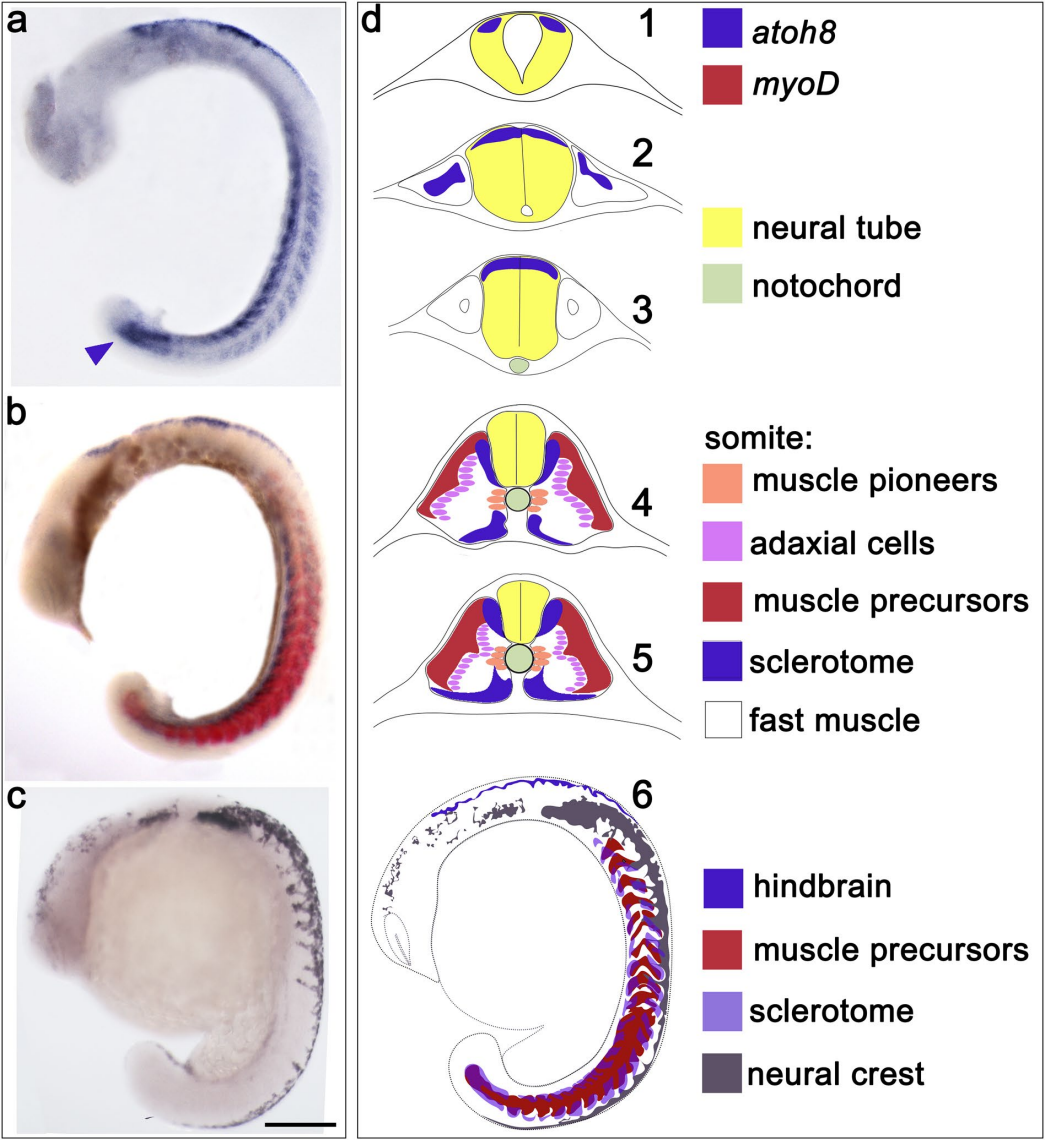
**a**–**c** Whole embryos at 18 hpf showing *atoh8, atoh8/myoD* and *crestin* expression, respectively; lateral view, anterior to the top. Arrowhead in a marks the chordoneural hinge. **d** Schematic overview showing distribution of cell and tissue types from anterior (top) to posterior positions in the embryo (**d1**–**d5**) and in the whole embryo viewed from laterally (**d6**). Scale bar 200 µm

**Fig. 5:**
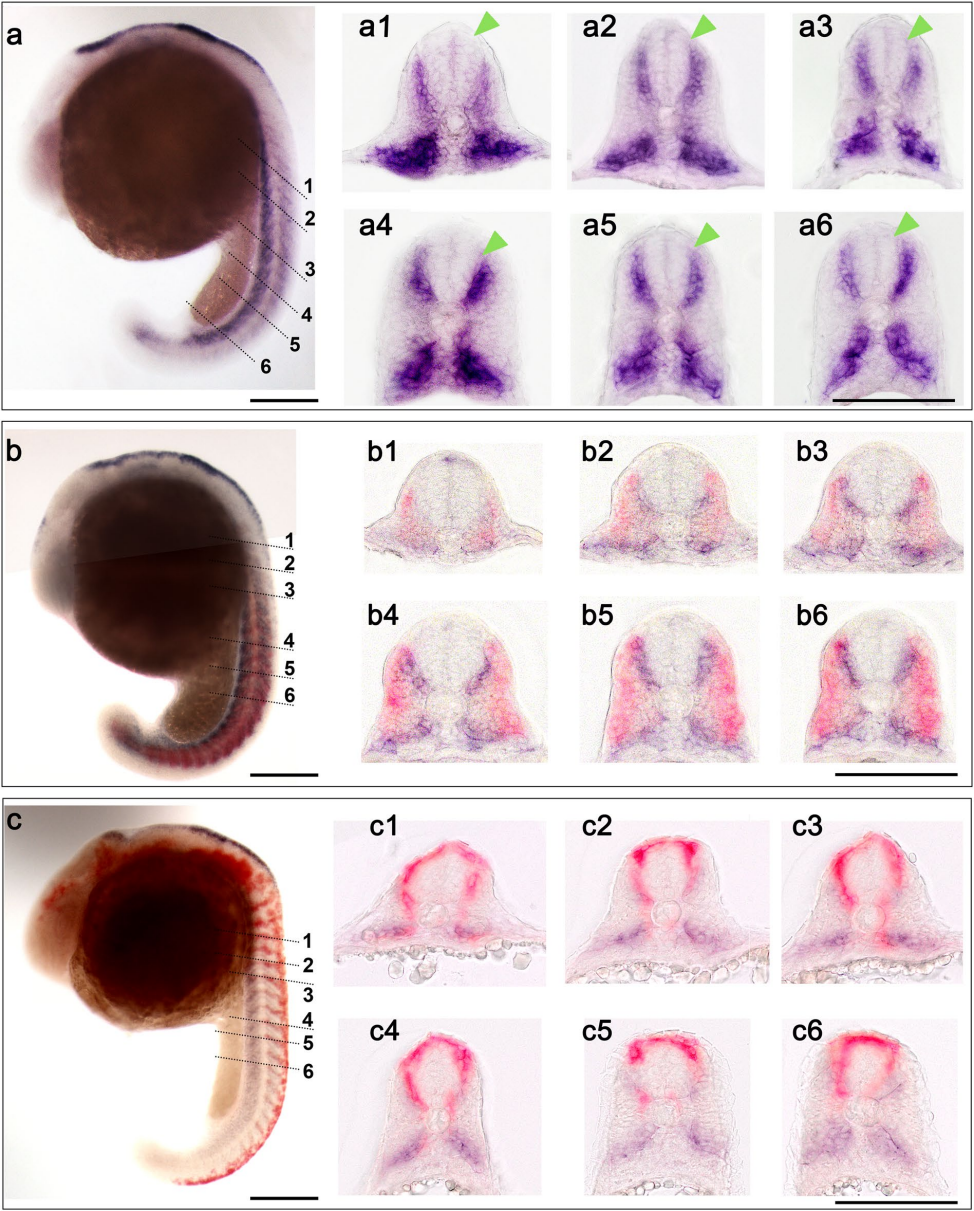
*atoh8*, *atoh8/myoD* and *atoh8/crestin* expression in whole embryos at 19–20 hpf (lateral view, dorsal to the right) and transverse sections of **a**, **b** and **c** at levels indicated. Green arrowheads in **a1**–**a6** indicate the position of neural crest cells. Scale bars: whole embryos 200 µm, transverse sections 100 µm

**Fig. 6:**
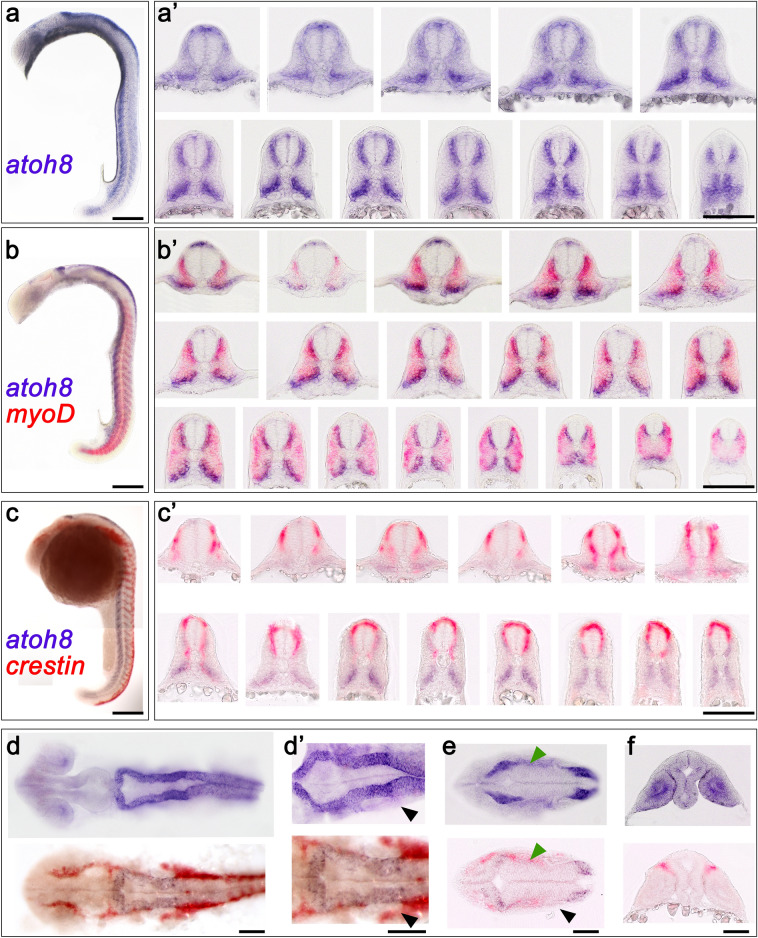
*atoh8*, *atoh8/myoD* and *atoh8/crestin* expression profile at 22 hpf. **a**–**c** Whole mounts (lateral view, dorsal to the right) and associated serial transverse sections. **d**, **d’** Flat mounts of zebrafish embryo showing expression in hindbrain, cerebellum and eye (dorsal view, anterior to the left). **e** Longitudinal sections of the hindbrain and cerebellum reveal the position of *atoh8* signal in the superficial layer of the developing neural tissue; *atoh8*-positive streams of cells can be detected adjacent to the otic vesicle (black arrowhead); there is no clarity about the tissue type of these cells, since they seem to reside in areas apparently marked by *crestin* transcription (green arrowheads). **f** Transverse section through the diencephalon demonstrating *atoh8* in the lens primordium and part of the retina (upper panel), and crestin at the temporal margin of the developing eye (lower panel). Scale bars: whole embryos 200 µm, flat mounts 100 µm, transverse sections 100 µm

**Fig. 7:**
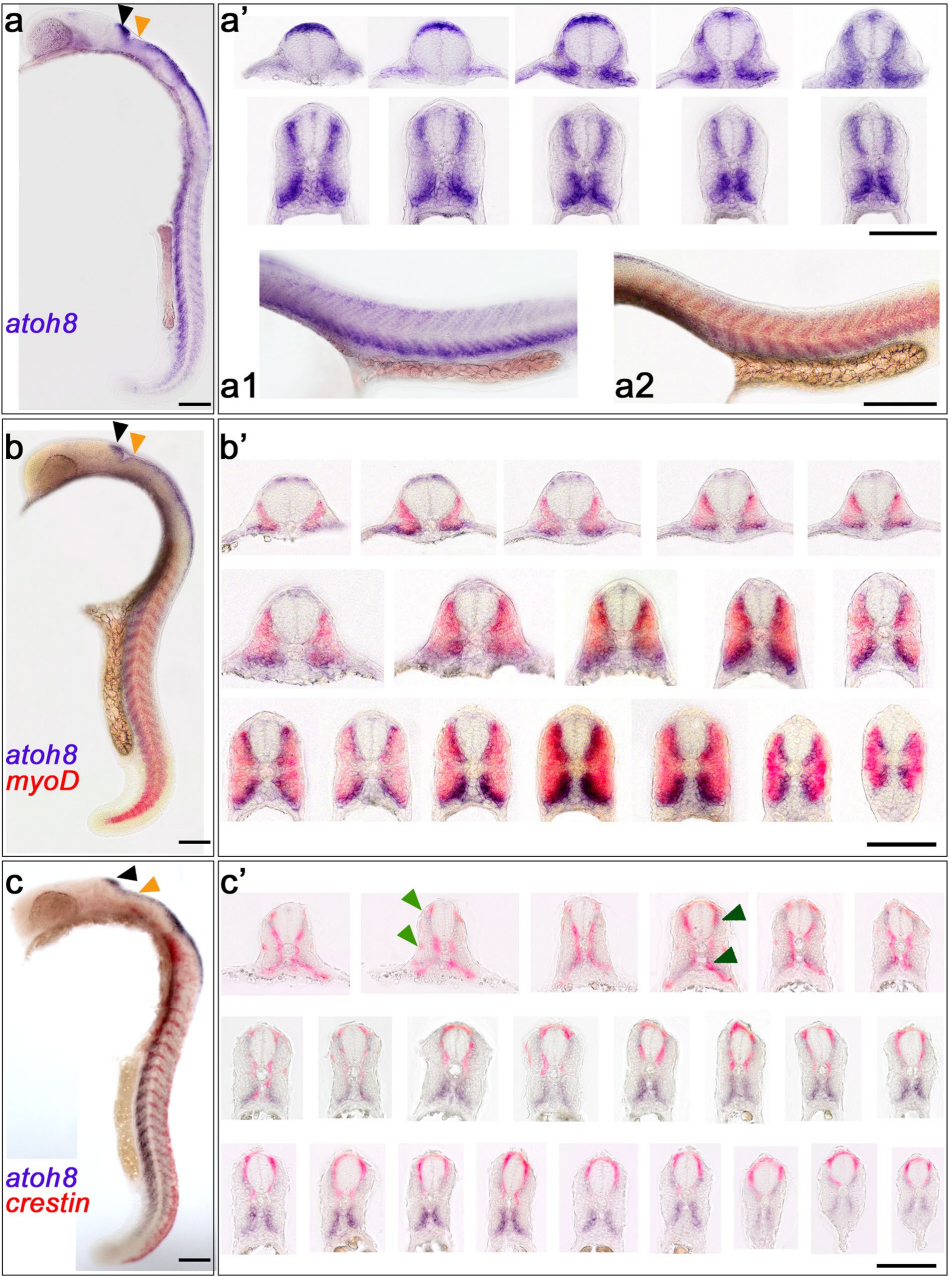
*atoh8*, *atoh8/myoD* and *atoh8/crestin* expression at 24 hpf. **a**–**c** Whole mounts (lateral view, dorsal to the right) and **a’**, **b’**, **c’** associated serial transverse sections. **a1**, **a2** Flat mounts of zebrafish trunk; lateral view, anterior to the left. *atoh8* is located in the deeper tissue of the somite, whereas *myoD* resides in the more superficial layers. Arrowheads in **a**, **b** and **c** pointing to hindbrain domains with strong (black arrowhead) and diminished (orange arrowhead) *atoh8* signal. Arrowheads in (**c’**) pointing to neural crest cells of the lateral (light green) and medial (dark green) migratory pathway. Scale bars: whole embryos 200 µm, flat mounts 200 µm, transverse sections 100 µm

In the first 24 h of zebrafish development, *atoh8* expression could be separated into two different phases. The initial phase, which lasted from about 9 hpf to 16 hpf, was characterised by the emergence of two broad and distinct domains of *atoh8* signal (Figs. 1, 2, 3). The first domain formed in the paraxial mesoderm, the second in precursor tissue of the embryonic brain. The second phase, lasting up to 24 hpf (Figs. 4, 5, 6, 7), showed a stable continuation of spatial *atoh8* expression in the previously established domains.

### *atoh8* expression starts at 9 hpf in the pre-segmental paraxial mesoderm

Contrary to previously published observations on *atoh8* expression (Yao et al. 2010), we detected an *atoh8* signal not earlier than about 9 hpf. The 6-hpf embryo was free of *atoh8* transcripts (Fig. 1a), as were the subsequent stages, before the first signal appeared in the paraxial mesodermal layer of the hypoblast (Fig. 1b) at around 9 hpf. Ventrally, the expression domain extended uniformly within the lower quarter of the paraxial mesoderm of the hypoblast (Fig. 1b’). Dorsally, it occupied a smaller, more defined location in the paraxial mesodermal layer with strongest expression at the level where later the first somite will emerge (Fig. 1b’’).

### *atoh8*, *myoD* and *crestin* expression in comparison

We noticed that the expression of *atoh8* in the early stages in our analysis differed from previously reported observations; therefore, we investigated, using double in situ hybridisation, the location of *myoD* and *crestin* transcripts in relation to *atoh8*.

The earliest expression of *myoD* was detected at about 7–7.5 hpf, in accordance with the known onset of *myoD* transcription, in a triangular patch of cells bilaterally positioned next to the embryonic shield (Weinberg et al. 1996), and during the next to 2–3 h of development, *myoD* expression extended anteriorly as a longitudinal row of cells on both sides adjacent to the axial mesoderm of the hypoblast (Fig. 1c). By 9.5 hpf, the dorsal and ventral *atoh8* expression domains started extending posteriorly and anteriorly, respectively (Fig. 1d). At 10 hpf (Fig. 1e–e’), the tailbud has formed and consists of a ventrally derived posterior portion and a dorsally derived anterior portion. *atoh8* continued to be expressed in the ventrally derived portion of the tailbud, which later will contribute to the posterior portion of the forming trunk and tail (Kanki and Ho 1997). Of note, the two expression domains in the anterior and posterior tailbud remained separated by a stretch of tissue that showed very little or no labelling in the early stages of development (Fig. 1e’, white arrowhead). The ventral view of the 10-hpf embryo showed uniform distribution of *atoh8* extending medially to laterally in two to three layers of cells in the ventrally derived portion of the tailbud (arrowheads Fig. 1f, f’). The onset and increasing strength of *atoh8* expression was confirmed by real-time quantitative PCR for the stages of 2.5 hpf to 11 hpf (Fig. 1g). Dorsal expression of *atoh8* continued extending from the level of the presumptive first somite towards the tailbud (Fig. 1h, h’). Both ventrally and dorsally, the signal remained restricted to the paraxial mesoderm layer of the hypoblast. The *myoD* signal extended anteriorly to the border of the trunk and head while remaining restricted to the adaxial cells (Fig. 1h, h’). In contrast to *atoh8*, *myoD* was never detected in the ventrally derived portion of the tailbud. Shortly after 10 hpf, segmentation of the paraxial mesoderm becomes apparent with formation of the first somite furrow demarcating the posterior half of the first somite from the anterior half of the second somite (Kimmel et al. 1995). At 10.5 hpf, the dorsal *atoh8* signal continued extending posteriorly (Fig. 1i) and by 11.5 hpf, ventral and dorsal tailbud *atoh8* domains lay in close proximity to each other (Fig. 1j). *myoD* expression at this stage extended to several rows of cells along the dorsal to ventral axis posteriorly (Fig. 1j’). At 12 hpf, the ventral and dorsal *atoh8* domains expanded further towards each other in the trunk and tail (Fig. 1k–k’’). *myoD* became detectable in bands of cells lateral to the notochord. Viewed dorsally, *atoh8* expression was clearly seen in the paraxial mesoderm, whereas axial mesoderm structures (notochord), adaxial cells and the single layer of epithelial cells enclosing the mesenchymal paraxial mesoderm ventrally, dorsally and laterally remained free of *atoh8* signal (Fig. 1k’’). This pattern of expression restricted to the mesenchymal somite was more evident at slightly later stages as revealed by transverse sections of older embryos (see below). Posteriorly, the expression of *atoh8* showed the same pattern as before, with *atoh8* transcripts occupying the unsegmented paraxial mesoderm and the mesenchymal core of newly formed somites (Fig. 1k’’). In the more mature anterior somites, however, *atoh8* signal translocated to progressively medial positions next to the forming neural tube and axial vessels (Fig. 1l, l'). Bands of *myo*D transcripts occupied the dorsal border of the newly emerging posterior and already formed anterior somites (faint and strong bands, respectively, in Fig. 1l and l').

By 13 hpf, expression of *atoh8* transcripts appeared in the forming hindbrain (Fig. 2, and see below). In the 13.5-hpf flat mounts (Fig. 2a), the *atoh8*-negative region between the posterior paraxial mesoderm and the posterior body wall became most obvious. This stretch of tissue free of *atoh8* signal is most likely to be the region of undetermined multipotential midline progenitor cells which, post gastrulation, contribute to the formation of the notochord, floor plate and, in anamniotes, hypochord during ongoing anterior to posterior axis elongation (Henrique et al. 2015; Row et al. 2016). The posterior body wall region itself is positive for *atoh8*, and interestingly, this is the source of a further pool of undetermined bipotential neuromesodermal progenitor cells—the posterior body wall cells—contributing to the formation of the spinal cord, somites and vasculature during embryonic axis elongation (Row et al. 2016; Tzouanacou et al. 2009).

At the trunk level, the previously mentioned differential distribution of *atoh8* signal along the anterior to posterior axis became apparent (Fig. 2a), and transverse sections revealed the progressive location of transcripts towards more medial positions in the more mature somites (Fig. 2e1), while in the recently formed (Fig. 2e2) as well as in the forming somites (Fig. 2e3), *atoh8* expression diffusely distributed within the mesenchymal domain. As observed for the previous stages, the one-cell-thick epithelial layer of the somite remained free of *atoh8* transcripts as did the adaxial cells. With ongoing segmentation, a pattern of mutually exclusive expression of *atoh8* and *myoD* revealed itself: in the trunk region, the two expression domains were strictly separated from each other (Fig. 2f, g). This became most obvious in the flat mounts of double-stained 13-hpf embryos (Fig. 2h, h’). While *myoD* expression started at the posterior margin, *atoh8* transcripts retracted from the posterior border and accumulated in the anterior half of the forming somite. Interestingly, *myoD* expression seemed to commence in the epithelial cells of the posterior somite border which where devoid of *atoh8* signal. In the recently formed somite, *myoD* further extended anteriorly, while *atoh8* concentrated medially next to the presumptive spinal cord dorsally and throughout the mediolateral extent of the somite ventrally. In the more anterior and mature somites, the *myoD* expression domain assumed a triangular shape in the flat mounts (Fig. 2 h and h’), extending throughout the medial region of the somite, while tapering from posteriorly to anteriorly. Thus the changing pattern of *atoh8* and *myoD* expression indicated the progressing maturation of the somites (Fig. 2g and h).

*crestin* expression followed the previously reported pattern starting at about 11 hpf (Luo et al. 2001). At 13 hpf and 14 hpf, cranial neural crest cells started migrating anteriorly towards the eye and ventrally along the margins of the otice vesicle (Fig. 2). We did not observe any overlap of *crestin* expression with *atoh8* at these stages. The two domains were distinguishably separated from each other in the hindbrain (Fig. 2 i–k), as well as in the trunk and tail (Fig. 2j, k).

At 16 hpf, *atoh8* signal was detectable in three distinct locations along the anterior to posterior axis of the embryo: anteriorly, in the head, in the posterior portion of the developing eye and in the presumptive hindbrain and cerebellum (Fig. 3a, a1, a2, b, c, d; see below); in the trunk and tail, in segmented and unsegmented paraxial mesoderm, and posteriorly in the anterior portion of the tail. *atoh8* transcripts started clearing from the most posteriorly located tailbud (Fig. 3a, 3b, 3e).

*atoh8* and *myoD* expression in the trunk and tail at this stage reflected very well the separation of the sclerotomal versus myotomal compartment in the progressively maturing somite. *atoh8* expression moved closer to the medial regions in the dorsal somite while remaining confined to the previously established pattern of mediolateral extension in the ventral somite (Fig. 3a 3–6). *myoD* expression translocated towards the superficial layers with advancing maturation of the somites (Fig. 3e, 3e1–3). At more posterior levels, *atoh8* expression persisted in the unsegmented paraxial mesoderm and in the emerging tail (Fig. 3e). As observed at previous stages, notochord and adaxial cells remained free of *atoh8* signal, while *myoD* transcripts were detectable in the adaxial cells (Fig. 3e2 and e3). At all trunk levels, *atoh8* signal was never detected within or above the developing neural tube (Fig. 3a, 3a3–6, 3b, 3e1–3).

*crestin* signal did not overlap with *atoh8* signal in the 16-hpf embryos (Fig. 3f–h). While *crestin* occupied areas at the lateral border of the neural plate in the hindbrain and above the neural keel/tube in the trunk and tail, *atoh8* was located in the neuroepithelium of the hindbrain and in the somites in the trunk and tail (Fig. 3f, g). Single in situ hybridisation with digoxigenin-labelled *crestin* mRNA resulted in a much stronger signal and clearly revealed that the expression domain of *crestin* in the brain, and especially the hindbrain, did not overlap with expression domain of *atoh8* (Fig. 3h).

### *atoh8* expression from 18 to 24 h of development

*atoh8* continued to be expressed in the stably generated major domains in the head, trunk and tail of the embryo from 18 until 24 hpf, the latest embryonic time point we analysed in detail. In addition, previously unlabelled cells in the head region laterally to the hindbrain start and maintain expression during this time period.

Lateral view of the 18-hpf embryos (Fig. 4a and b) made apparent the characteristic L shape of the ventral sclerotome as described in the original study (Morin-Kensicki and Eisen 1997). *atoh8* transcripts were also detectable in the tail in paraxial mesoderm surrounding the chordoneural hinge (Fig. 4a, arrowhead), which itself remained free of signal. *myoD* expression declined in the most anterior somites, while remaining strong more posteriorly in the trunk and in the tail (Fig. 4b).

*atoh8* was located in the uppermost layer of the neuroepithelium of the developing cerebellum (Fig. 4d1) and the hindbrain at the level anteriorly and posteriorly to the otic vesicle (Fig. 4d2). At this levels, atoh8 transcripts were located also in tissue next to the neural tube. Because of failure to retrieve transverse sections at these levels in the double-stained 18-hpf embryos, we could not determine whether or not there was an overlap of *atoh8* and *crestin* signal in this area. More posteriorly, *atoh8* signal was again restricted to the uppermost layer of the neural tube (Fig. 4d3).

In the anterior somites, the initially cuboidal adaxial cells elongate and commit to the slow fibre type. While those fibres at the dorsoventral midline remain next to the notochord and differentiate into muscle pioneers, the remaining fibres migrate radially through the somite to progressively lateral positions (Fig. 4d4 and d5), eventually forming a superficial single layer of mononucleated slow muscle fibres (Cortés et al. 2003; Devoto et al. 1996; Gurevich et al. 2015). Migration of the adaxial cells leaves behind a butterfly-shaped domain of medial fast fibre precursors void of *myoD* signal, without affecting the position of *atoh8* transcription in the dorsal and ventral domain (Fig. 4d4 and d5). *crestin* signal expanded posteriorly and, in distinct streams along the anterior–posterior midline of the somites, ventrally in the upper trunk region (Fig. 4c, d6). The schematic drawing in Fig. 4d6 illustrates the distribution of the three distinct domains in the 18-hpf embryo.

At 20 hpf, hindbrain, cerebellum, trunk and tail showed expression domains as detected before (Fig. 5). Transverse sections displayed the characteristic distribution of *atoh8* transcripts at the trunk levels (Fig. 5a, a1–6). As observed in previous stages, cells at locations where neural crest cells were expected to reside remain free of *atoh8* signal (exemplified in Fig. 5a1–a6, green arrowheads). *myoD* and *atoh8* domains did not overlap (Fig. 5b, b1–6), neither did *atoh8* and *crestin* domains (Fig. 5c, c1-6).

In the embryo at 22 hpf, a continuation of the established expression pattern of the three transcription factors was observable (Fig. 6). The mutual exclusiveness of the three expression domains was once again confirmed in serial transverse sections through the trunk of the embryo (Fig. 6a’, b’ and c’). Flat mounts of embryos demonstrated well the separation of *crestin*-labelled neural crest cells and *atoh8*-labelled neuroepithelium (Fig. 6d and d’). The location of the *atoh8* signal in the uppermost layer of the hindbrain neuroepithelium was confirmed by coronal sections in the deeper levels of the embryonic head (Fig. 6e). However, there was ambiguity about co-expression of *atoh8* and *crestin* at this level (Fig. 6e, green arrowheads).

Although *atoh8* signal appeared weaker in the dorsal than in the ventral trunk and tail region in whole mount embryos of 24 hpf (Fig. 7), serial transverse sections did not reveal a pattern significantly different between dorsal and ventral expression domains from that in previous stages (Fig. 7a’). Flat mounts of the zebrafish trunk demonstrated that *atoh8* is located in the deeper tissue of the somite (Fig. 7a1 and a2), whereas *myoD* resides in the more superficial layers (Fig. 7a2). *myoD* expression followed the previously described anterior to posterior pattern of distribution in the forming myotome (Weinberg et al. 1996). Serial transverse sections (Fig. 7b’) once again revealed the mutual exclusion of *atoh8* and *myoD* expression in the forming myotome, especially in the more posterior sections. As with *myoD*, *crestin* did not show spatial overlap with *atoh8* in its expression (Fig. 7 and c’). At this stage, *crestin* marked cells of the lateral and medial migratory pathway destined to become melanocytes and dorsal root and sympathetic chain ganglia, respectively (exemplified in Fig. 7c’ by green arrowheads).

## Discussion

We identified *atoh8* expression in well-defined regions of the presumptive hindbrain and cerebellum, and sclerotome of the early embryonic zebrafish. These results are in contrast to previously reported domains of *atoh8* expression in zebrafish (Place and Smith 2017; Rawnsley et al. 2013; Fang et al. 2014; Yao et al. 2010).

### *atoh8* and *crestin* expression occurs in different domains

Our double in situ expression analysis excluded the co-expression of *crestin* and *atoh8.* In zebrafish, neural crest progenitors emerge from about 10.5 hpf at the lateral margin of the neural ectoderm and begin their migration in well-defined streams at about 13 hpf (cranial) and 15 hpf (trunk) (Odenthal and Nüsslein-Volhard 1998; Raible et al. 1992; Rocha et al. 2020; Schilling and Kimmel 1994; Thisse et al. 1995). We never found *atoh8*-positive cells located adjacent to the lateral border of the neural epithelium, neither in the rhombencephalic nor in the trunk and tail region. *atoh8* signal remained always restricted to the superficial layers of the hindbrain and within the pre-segmental paraxial mesoderm and the forming and formed somites. We never observed *atoh8* labelling in cells residing above the neural tube (pre-migratory trunk neural crest) nor in cells migrating between the somites and neural tube (migratory neural crest on their medial pathway) or cells migrating between somites and overlying ectoderm (lateral pathway). Although, at later stages, cells were observed between the neural tube and the *atoh8*-positive sclerotome at positions where neural crest derivatives as, for example, dorsal rot ganglia, are located in the zebrafish trunk, these cells remained *atoh8*-negative (Fig. 5a, green arrowheads and c). The static expression pattern of *atoh8* and its restriction to an inner-somitic domain strongly argues against the notion that *atoh8* is labelling the trunk neural crest and supports the idea of *atoh8* labelling the sclerotome compartment in the somites. We cannot maintain this strong argument for the population of cells staining *atoh8*-positive in the head region lateral to the neuroepithelium (Fig. 6e, green arrowheads); however, we favour the possibility of the stained cells being of sclerotomal origin, since some of the skeletal craniofacial structures in zebrafish are known to arise not from the neural crest but from the mesoderm (Kague et al. 2012; Yelick and Schilling 2002).

### *atoh8* presumably marks developing hindbrain and cerebellum

The domain defined by *atoh8* expression in the hindbrain and presumptive cerebellum has been demonstrated to be the area of subsets of cells that express the zebrafish atonal homologues *atoh1a*, *atoh1b* and *atoh1c* and are known to contribute to the generation and specification of neuronal derivatives in the hindbrain and cerebellum (Adolf et al. 2004; Kani et al. 2010; Kidwell et al. 2018; Volkmann et al. 2010). In the mouse, *math6* has been shown to be expressed in the developing cerebellum, promoting the differentiation of precursors towards the neural versus the glial fate (Inoue et al. 2001). Considering the expression pattern of the other atonal genes, therefore, it is possible that *atoh8* is complementing this set of bHLH genes. Of course, this remains to be established by further in situ expression analysis and gene expression manipulation.

### *atoh8* and *myoD* expression domains do not overlap

As shown by our double expression analysis, sclerotome and myotome precursors separate from each other by means of their expression profiles from the emergence of paraxial mesoderm. *myoD*, as the first muscle-specific marker to be expressed, resides in areas within the somite which are distinct from those occupied by *atoh8*. Thus, *atoh8* does not seem to mark cells contributing to the developing myotome. Both *atoh8* and *myoD* maintain their characteristic and distinct expression profiles throughout the developmental stages analysed. A second wave of *myoD* expression occurs from 30 hpf in the fin primordia and in the muscles of the jaw and eye, and *myoD* continues being expressed in the myotome up until at least 60 hpf (Weinberg et al. 1996), long after the first phase of muscle development in the trunk has been completed. We started observing *atoh8* in the non-neural head region from about 18 hpf in cells we believe to be of mesodermal origin. These cells later might contribute to components of the neurocranium and viscerocranium and to the fin buds derived from the sclerotome. We observed *atoh8* staining from 19 hpf in the fin bud primordium until 72 hpf (our own unpublished observations). It is conceivable that *atoh8* in these structures is being expressed in advance of *myoD*, since in all developmental stages analysed, *atoh8* expression preceded *myoD* expression in the paraxial mesoderm with the exception of the adaxial cells.

### *atoh8* marks the sclerotomal compartment of the developing somite

The sclerotomal *atoh8*-positive subpopulation of cells in zebrafish establishes itself quite early in development, much like the subpopulation of cells destined to form muscle tissue, i.e., even before overt morphological segmentation (Fig. 2h). Furthermore, the sclerotome does not seem to originate from the epithelial layer of cells in the somite (Fig. 2 e). This is in contrast to sclerotome in amniotes, as in chick and mice, the sclerotome ensues from the ventral half of the epithelial somite (Christ et al. 2004; Monsoro-Burq 2003; Tajbakhsh and Spörle 1998).

The initial phase of *atoh8* expression, in our view, shows the generation of the sclerotomal compartment of the somite. The steady continuation of *atoh8* expression in the second phase supports our assumption of *atoh8* marking the sclerotome in the somite. The areas associated with *atoh8* expression have been shown to be positive for other markers such as *twist* (Germanguz et al. 2007; Gitelman 2007; Yeo et al., 2007, 2009), *pax* (Liu et al. 2020; Ma et al. 2018; Nornes et al. 1996) and *nkx3* (Ma et al. 2018), all genes which are known to be specifically expressed in the sclerotome of vertebrates. Our expression profile of *atoh8* furthermore corroborates a previously observed finding (Ma et al. 2018), which points to a novelty regarding the established location of the vertebrate sclerotome: while in amniotes the sclerotome is restricted to a ventral domain from which later on in development cells migrate along the notochord to dorsal positions, there exists a further, dorsal domain in zebrafish (Ma et al. 2018). Interestingly, we also found *atoh8* to be expressed in this dorsal domain in addition to the canonical ventral domain. In our study, *atoh8* marked the dorsal and ventral sclerotome domains at the same time from their emergence, and we did not detect *atoh8*-positive cells adjacent to the notochord at any stage in our expression study. There are two conceivable explanations for this: *atoh8* might be expressed in only the ventral and dorsal subpopulation of sclerotome cells and does not mark the subpopulation of notochord-associated cells or it is expressed only in non-migratory precursor cells.

In conclusion, we cannot corroborate previous findings of expression of *atoh8* in the zebrafish neural crest and myotome (Yao et al. 2010). All *myoD*-positive cells in the somite and myotome and all *crestin*-positive cells in the head and trunk did not co-localise with *atoh8* at any of the observed stages of development. Basically, throughout the observed stages of development, the expression domains co-existed but remained spatially separated. Thus, *atoh8*, *crestin* and *myoD* clearly mark mutually exclusive expression domains in the early zebrafish embryo.

Although *atoh8* is not expressed in muscle progenitor cells, the finding that knock-down disrupts the coherence of muscle tissue (Yao et al. 2010) is not contradictory. The sclerotome gives rise to tendons and ligaments of the axial skeleton in zebrafish (Ma et al. 2018). These structures play a crucial role in enabling the transmission of force during movement from very early stages of development in zebrafish. Thus, they have to be established concomitant with the muscular system prior to the formation of cartilage and bone, which, in zebrafish, does not develop before the larval stages. Disruption of pathways involved in sclerotome formation and/or maturation, therefore, inevitably affects the structural and functional integrity of muscle tissue. In light of this, the simultaneous spatial expression of *atoh8* and *myoD* in mutually exclusive domains is consistent with different, but possibly interdependent roles of these two transcription factors in zebrafish somite development.

While the morphant phenotype of *atoh8* knock-down might be explained via the subtle regulatory interplay of the myotome and sclerotome, there still needs to be investigated the lack of an obvious phenotype of the *atoh8* knock-out. In view of the discrepancies regarding the expression of *atoh8* in various tissue types and the varying effects of gene manipulation or lack thereof, however, it is conceivable that a subtle knock-out phenotype has not been discovered yet due to the fact that *atoh8* is not expressed in the tissues and organs investigated so far in zebrafish.

In summary, our study highlights for the first time the exact spatio-temporal course of *atoh8* expression in the first 24 h of zebrafish development and reveals a profile that is clearly restricted to sclerotome as opposed to myotome in the somite. Its expression domains in the cranial regions of the zebrafish suggests involvement of *atoh8* in the development of the hindbrain and cerebellum. The static pattern of expression restricted to well-defined regions in the head and trunk argues against the notion of *atoh8* labelling neural crest cells. We believe our findings facilitate and encourage further studies into sclerotome formation in zebrafish and serve to elucidate and advance investigations into possible roles of *atoh8* in zebrafish development.

## Data Availability

All data and materials generated/used in this study are available on request.

## References

[CR1] Adolf B, Bellipanni G, Huber V, Bally-Cuif L (2004). atoh1.2 and beta3.1 are two new bHLH-encoding genes expressed in selective precursor cells of the zebrafish anterior hindbrain. Gene Expr Patterns.

[CR2] Akazawa H, Komuro I, Sugitani Y, Yazaki Y, Nagai R, Noda T (2000). Targeted disruption of the homeobox transcription factor Bapx1 results in lethal skeletal dysplasia with asplenia and gastroduodenal malformation. Gene Cells.

[CR3] Balling R, Neubüser A, Christ B (1996). Pax genes and sclerotome development. Sem Cell Dev Biol.

[CR4] Barnes RM, Firulli AB (2009). A twist of insight-the role of Twist-family bHLH factors in development. Int J Dev Biol.

[CR5] Belzunce I, Belmonte CM, Corbi PC (2020). The interplay of atoh1 genes in the lower rhombic lip during hindbrain morphogenesis. PLoS ONE.

[CR6] Bénazéraf B, Pourquié O (2013). Formation and segmentation of the vertebrate body axis. Annu Rev Cell Dev Biol.

[CR7] Bensimon-Brito A, Cardeira J, Cancela ML, Huysseune A, Witten PE (2012). Distinct patterns of notochord mineralization in zebrafish coincide with the localization of Osteocalcin isoform 1 during early vertebral centra formation. BMC Dev Bio.

[CR8] Brand-Saberi B, Wilting J, Ebensperger C, Christ B (1996). The formation of somite compartments in the avian embryo. Int J Dev Biol.

[CR9] Chen X, Huang H, Wang H, Guo F, Du X, Ma L, Zhao L, Pan Z, Gui H, Yuan T, Liu X, Song L, Wang Y, He J, Lei H, Gao R (2014). Characterization of zebrafish Pax1b and Pax9 in fin bud development. Biomed Res Int.

[CR10] Christ B, Huang R, Scaal M (2004). Formation and differentiation of the avian sclerotome. Anat Embryol.

[CR11] Cortés F, Daggett D, Bryson-Richardson RJ, Neyt C, Maule J, Gautier P, Hollway GE, Keenan D, Currie PD (2003). Cadherin-mediated differential cell adhesion controls slow muscle cell migration in the developing zebrafish myotome. Dev Cell.

[CR12] Devoto SH, Melançon E, Eisen JS, Westerfield M (1996). Identification of separate slow and fast muscle precursor cells in vivo, prior to somite formation. Development.

[CR13] Fang F, Wasserman SM, Torres-Vazquez J, Weinstein B, Cao F, Li Z, Wilson KD, Yue W, Wu JC, Xie X, Pei X (2014). The role of Hath6, a newly identified shear-stress-responsive transcription factor, in endothelial cell differentiation and function. J Cell Sci.

[CR14] Fleming A, Keynes RJ, Tannahill D (2001). The role of the notochord in vertebral column formation. J Anat.

[CR15] Fleming A, Keynes R, Tannahill D (2004). A central role for the notochord in vertebral patterning. Development.

[CR16] Fleming A, Kishida MG, Kimmel CB, Keynes RJ (2015). Building the backbone: the development and evolution of vertebral patterning. Development.

[CR17] Germanguz I, Lev D, Waisman T, Kim CH, Gitelman I (2007). Four twist genes in zebrafish, four expression patterns. Dev Dyn.

[CR18] Gitelman I (1997). Twist protein in mouse embryogenesis. Dev Biol.

[CR19] Gitelman I (2007). Evolution of the vertebrate twist family and synfunctionalization: a mechanism for differential gene loss through merging of expression domains. Mol Biol Evol.

[CR20] Gridley T (2006). The long and short of it: somite formation in mice. Dev Dyn.

[CR21] Gurevich D, Siegel A, Currie PD (2015). Skeletal myogenesis in the zebrafish and its implications for muscle disease modelling. Vertebrate MYOGENESIS.

[CR22] Henrique D, Abranches E, Verrier L, Storey KG (2015). Neuromesodermal progenitors and the making of the spinal cord. Development.

[CR23] Herbrand H, Pabst O, Hill R, Arnold H-H (2002). Transcription factors Nkx3.1 and Nkx3.2 (Bapx1) play an overlapping role in sclerotomal development of the mouse. Mech Dev.

[CR24] Inohaya K, Takano Y, Kudo A (2007). The teleost intervertebral region acts as a growth center of the centrum: in vivo visualization of osteoblasts and their progenitors in transgenic fish. Dev Dyn.

[CR25] Inoue C, Bae SK, Takatsuka K, Inoue T, Bessho Y, Kageyama R (2001). Math6, a bHLH gene expressed in the developing nervous system, regulates neuronal versus glial differentiation. Gene Cells.

[CR26] Kague E, Gallagher M, Burke S, Parsons M, Franz-Odendaal T, Fisher S (2012). Skeletogenic fate of zebrafish cranial and trunk neural crest. PLoS ONE.

[CR27] Kani S, Bae Y-K, Shimizu T, Tanabe K, Satou C, Parsons MJ, Scott E, Higashijima S-i, Hibi M (2010). Proneural gene-linked neurogenesis in zebrafish cerebellum. Dev Biol.

[CR28] Kanki JP, Ho RK (1997). The development of the posterior body in zebrafish. Development.

[CR29] Keenan SR, Currie PD (2019). The developmental phases of zebrafish myogenesis. J Dev Biol.

[CR30] Kidwell CU, Su C-Y, Hibi M, Moens CB (2018). Multiple zebrafish atoh1 genes specify a diversity of neuronal types in the zebrafish cerebellum. Dev Biol.

[CR31] Kiecker C, Lumsden A (2005). Compartments and their boundaries in vertebrate brain development. Nat Rev Neurosci.

[CR32] Kimmel CB, Ballard WW, Kimmel SR, Ullmann B, Schilling TF (1995). Stages of embryonic development of the zebrafish. Dev Dyn.

[CR33] Liu X, Wang H, Li G, Huang HZ, Wang YQ (2013). The function of DrPax1b gene in the embryonic development of zebrafish. Genes Genet Syst.

[CR34] Liu Y-H, Lin T-C, Hwang S-PL (2020). Zebrafish Pax1a and Pax1b are required for pharyngeal pouch morphogenesis and ceratobranchial cartilage development. Mech Dev.

[CR35] Luo R, An M, Arduini BL, Henion PD (2001). Specific pan-neural crest expression of zebrafish Crestin throughout embryonic development. Dev Dyn.

[CR36] Ma RC, Jacobs CT, Sharma P, Kocha KM, Huang P (2018). Stereotypic generation of axial tenocytes from bipartite sclerotome domains in zebrafish. PLoS Genet.

[CR37] Maroto M, Bone RA, Dale JK (2012). Somitogenesis. Development.

[CR38] Millimaki BB, Sweet EM, Dhason MS, Riley BB (2007). Zebrafish atoh1 genes: classic proneural activity in the inner ear and regulation by Fgf and Notch. Development.

[CR39] Monsoro-Burq A-H (2003). Sclerotome development and morphogenesis: when experimental embryology meets genetics. Int J Dev Biol.

[CR40] Morin-Kensicki EM, Eisen JS (1997). Sclerotome development and peripheral nervous system segmentation in embryonic zebrafish. Development.

[CR41] Morin-Kensicki EM, Melancon E, Eisen JS (2002). Segmental relationship between somites and vertebral column in zebrafish. Development.

[CR42] Nguyen-Chi ME, Bryson-Richardson R, Sonntag C, Hall TE, Gibson A, Sztal T, Chua W, Schilling TF, Currie PD (2012). Morphogenesis and cell fate determination within the adaxial cell equivalence group of the zebrafish myotome. PLoS Genet.

[CR43] Nornes S, Mikkola I, Krauss S, Delghandi M, Perander M, Johansen T (1996). Zebrafish Pax9 encodes two proteins with distinct C-terminal transactivating domains of different potency negatively regulated by adjacent N-terminal sequences. J Biol Chem.

[CR44] Odenthal J, Nüsslein-Volhard C (1998). *fork head* domain genes in zebrafish. Dev Genes Evol.

[CR45] Peters H, Wilm B, Sakai N, Imai K, Maas R, Balling R (1999). Pax1 and Pax9 synergistically regulate vertebral column development. Development.

[CR46] Place ES, Smith JC (2017). Zebrafish atoh8 mutants do not recapitulate morpholino phenotypes. PLoS ONE.

[CR47] Provot S, Kempf H, Murtaugh LC, Chung U-i, Kim D-W, Chyung J, Kronenberg HM, Lassar AB (2006). Nkx3.2/Bapx1 acts as a negative regulator of chondrocyte maturation. Development.

[CR48] Raible DW, Wood A, Hodsdon W, Henion PD, Weston JA, Eisen JS (1992). Segregation and early dispersal of neural crest cells in the embryonic zebrafish. Dev Dyn.

[CR49] Rainbow RS, Kwon H, Zeng L (2014). The role of Nkx3.2 in chondrogenesis. Front Biol.

[CR50] Rawnsley DR, Xiao J, Lee JS, Liu X, Mericko-Ishizuka P, Kumar V, He J, Basu A, Lu M, Lynn FC (2013). The transcription factor Atonal homolog 8 regulates Gata4 and Friend of Gata-2 during vertebrate development. J Biol Chem.

[CR51] Renn J, Büttner A, To TT, Chan SJH, Winkler C (2013). A col10a1: nlGFP transgenic line displays putative osteoblast precursors at the medaka notochordal sheath prior to mineralization. Dev Biol.

[CR52] Rocha M, Singh N, Ahsan K, Beiriger A, Prince VE (2020). Neural crest development: insights from the zebrafish. Dev Dyn.

[CR53] Row RH, Tsotras SR, Goto H, Martin BL (2016). The zebrafish tailbud contains two independent populations of midline progenitor cells that maintain long-term germ layer plasticity and differentiate in response to local signaling cues. Development.

[CR54] Rubinstein AL, Lee D, Luo R, Henion PD, Halpern ME (2000). Genes dependent on zebrafish cyclops function identified by AFLP differential gene expression screen. Genesis.

[CR55] Saga Y (2012). The mechanism of somite formation in mice. Curr Opin Genet Dev.

[CR56] Schilling TF, Kimmel CB (1994). Segment and cell type lineage restrictions during pharyngeal arch development in the zebrafish embryo. Development.

[CR57] Stellabotte F, Dobbs-McAuliffe B, Fernández DA, Feng X, Devoto SH (2007). Dynamic somite cell rearrangements lead to distinct waves of myotome growth. Development.

[CR58] Stickney HL, Barresi MJ, Devoto SH (2000). Somite development in zebrafish. Dev Dyn.

[CR59] Stockdale FE, Nikovits W, Christ B (2000). Molecular and cellular biology of avian somite development. Dev Dyn.

[CR60] Tajbakhsh S, Spörle R (1998). Somite development: constructing the vertebrate body. Cell.

[CR61] Tani S, Chung U-i, Ohba S, Hojo H (2020). Understanding paraxial mesoderm development and sclerotome specification for skeletal repair. Exp Mol Med.

[CR62] Terriente J, Gerety SS, Watanabe-Asaka T, Gonzalez-Quevedo R, Wilkinson DG (2012). Signalling from hindbrain boundaries regulates neuronal clustering that patterns neurogenesis. Development.

[CR63] Thisse B, Thisse C (2014). In situ hybridization on whole-mount zebrafish embryos and young larvae. In situ hybridization protocols.

[CR64] Thisse C, Thisse B, Schilling T, Postlethwait J (1993). Structure of the zebrafish snail1 gene and its expression in wild-type, spadetail and no tail mutant embryos. Development.

[CR65] Thisse C, Thisse B, Postlethwait JH (1995). Expression ofsnail2, a second member of the zebrafish snail family, in cephalic mesendoderm and presumptive neural crest of wild-type andspadetail mutant embryos. Dev Biol.

[CR66] Tribioli C, Lufkin T (1999). The murine Bapx1 homeobox gene plays a critical role in embryonic development of the axial skeleton and spleen. Development.

[CR67] Tzouanacou E, Wegener A, Wymeersch FJ, Wilson V, Nicolas J-F (2009). Redefining the progression of lineage segregations during mammalian embryogenesis by clonal analysis. Dev Cell.

[CR68] Volkmann K, Rieger S, Babaryka A, Köster RW (2008). The zebrafish cerebellar rhombic lip is spatially patterned in producing granule cell populations of different functional compartments. Dev Biol.

[CR69] Volkmann K, Chen YY, Harris MP, Wullimann MF, Köster RW (2010). The zebrafish cerebellar upper rhombic lip generates tegmental hindbrain nuclei by long-distance migration in an evolutionary conserved manner. J Comp Neurol.

[CR70] Weinberg ES, Allende ML, Kelly CS, Abdelhamid A, Murakami T, Andermann P, Doerre OG, Grunwald DJ, Riggleman B (1996). Developmental regulation of zebrafish MyoD in wild-type, no tail and spadetail embryos. Development.

[CR71] Willems B, Büttner A, Huysseune A, Renn J, Witten PE, Winkler C (2012). Conditional ablation of osteoblasts in medaka. Dev Biol.

[CR72] Wullimann MF, Mueller T, Distel M, Babaryka A, Grothe B, Köster RW (2011). The long adventurous journey of rhombic lip cells in jawed vertebrates: a comparative developmental analysis. Front Neuroanat.

[CR73] Yang D-C, Tsai C-C, Liao Y-F, Fu H-C, Tsay H-J, Huang T-F, Chen Y-H, Hung S-C (2011). Twist controls skeletal development and dorsoventral patterning by regulating runx2 in zebrafish. PLoS ONE.

[CR74] Yao J, Zhou J, Liu Q, Lu D, Wang L, Qiao X, Jia W (2010). Atoh8, a bHLH transcription factor, is required for the development of retina and skeletal muscle in zebrafish. PLoS ONE.

[CR75] Yelick PC, Schilling TF (2002). Molecular dissection of craniofacial development using zebrafish. Crit Rev Oral Biol Med.

[CR76] Yeo G-H, Cheah FS, Jabs EW, Chong SS (2007). Zebrafish twist1 is expressed in craniofacial, vertebral, and renal precursors. Dev Genes Evol.

[CR77] Yeo GH, Cheah FS, Winkler C, Jabs EW, Venkatesh B, Chong SS (2009). Phylogenetic and evolutionary relationships and developmental expression patterns of the zebrafish twist gene family. Dev Genes Evol.

